# Association and Interaction Between Serum Interleukin-6 Levels and Metabolic Dysfunction-Associated Fatty Liver Disease in Patients With Severe Coronavirus Disease 2019

**DOI:** 10.3389/fendo.2021.604100

**Published:** 2021-03-08

**Authors:** Feng Gao, Kenneth I. Zheng, Hua-Dong Yan, Qing-Feng Sun, Ke-Hua Pan, Ting-Yao Wang, Yong-Ping Chen, Giovanni Targher, Christopher D. Byrne, Jacob George, Ming-Hua Zheng

**Affiliations:** ^1^Department of Gastroenterology, The First Affiliated Hospital of Wenzhou Medical University, Wenzhou, China; ^2^MAFLD Research Center, Department of Hepatology, The First Affiliated Hospital of Wenzhou Medical University, Wenzhou, China; ^3^Department of Hepatology, Key Laboratory of Diagnosis and Treatment of Digestive System Tumors of Zhejiang Province, Hwamei Hospital, Ningbo No.2 Hospital, University of Chinese Academy of Sciences, Ningbo, China; ^4^Department of Infectious Diseases, Ruian People’s Hospital, Wenzhou, China; ^5^Department of Radiology, The First Affiliated Hospital of Wenzhou Medical University, Wenzhou, China; ^6^Department of Nephrology, The First Affiliated Hospital of Wenzhou Medical University, Wenzhou, China; ^7^Section of Endocrinology, Diabetes and Metabolism, Department of Medicine, University and Azienda Ospedaliera Universitaria Integrata of Verona, Verona, Italy; ^8^Southampton National Institute for Health Research Biomedical Research Centre, University Hospital Southampton, Southampton General Hospital, Southampton, United Kingdom; ^9^Storr Liver Centre, Westmead Institute for Medical Research, Westmead Hospital and University of Sydney, Sydney, NSW, Australia; ^10^Institute of Hepatology, Wenzhou Medical University, Wenzhou, China; ^11^Key Laboratory of Diagnosis and Treatment for The Development of Chronic Liver Disease in Zhejiang Province, Wenzhou, China

**Keywords:** COVID-19, SARS-CoV-2, MAFLD, cytokine, IL-6, NAFLD

## Abstract

**Background and Aim:**

Circulating levels of interleukin (IL)-6, a well-known inflammatory cytokine, are often elevated in coronavirus disease-2019 (COVID-19). Elevated IL-6 levels are also observed in patients with metabolic dysfunction-associated fatty liver disease (MAFLD). Our study aimed to describe the association between circulating IL-6 levels and MAFLD at hospital admission with risk of severe COVID-19.

**Methods:**

A total of 167 patients with laboratory-confirmed COVID-19 from three Chinese hospitals were enrolled. Circulating levels of IL-2, IL-4, IL-6, IL-10, tumor necrosis factor (TNF)-α, and interferon (IFN)-γ were measured at admission. All patients were screened for fatty liver by computed tomography. Forty-six patients were diagnosed as MAFLD.

**Results:**

Patients with MAFLD (n = 46) had higher serum IL-6 levels (median 7.1 [interquartile range, 4.3–20.0] vs. 4.8 [2.6–11.6] pg/mL, *p* = 0.030) compared to their counterparts without MAFLD (n = 121). After adjustment for age and sex, patients with MAFLD had a ~2.6-fold higher risk of having severe COVID-19 than those without MAFLD. After adjustment for age, sex and metabolic co-morbidities, increased serum IL-6 levels remained associated with higher risk of severe COVID-19, especially among infected patients with MAFLD (adjusted-odds ratio 1.14, 95% CI 1.05–1.23; *p* = 0.002). There was a significant interaction effect between serum IL-6 levels and MAFLD for risk of severe COVID-19 (*p* for interaction = 0.008).

**Conclusions:**

Patients with MAFLD and elevated serum IL-6 levels at admission are at higher risk for severe illness from COVID-19.

## Introduction

The coronavirus disease 2019 (COVID-19) pandemic continues to attract worldwide attention. The occurrence of a virus-induced cytokine ‘storm’ is associated with greater disease severity and unfavorable in-hospital outcomes (1, 2). Elevated levels of serum interleukin-6 (IL-6) are a hallmark inflammatory signature commonly seen in patients with severe COVID-19 and the use of IL-6-receptor blocking antibodies has recently been approved in China for treatment of COVID-19 patients with serious pulmonary damage and elevated serum IL-6 levels (3).

Metabolic dysfunction-associated fatty liver disease (MAFLD), the newly proposed name for non-alcoholic fatty liver disease (NAFLD) (4), has reached epidemic proportions, affecting up to a quarter of the world’s adult population (5). MAFLD is known to be associated with various hepatic and extra-hepatic complications, such as cirrhosis, liver cancer, cardiovascular disease, type 2 diabetes and chronic kidney disease (6). We recently showed that younger patients with MAFLD have higher odds of severe COVID-19 (7).

Patients with MAFLD were characterized by impaired hepatic immunity (8, 9). Hepatic macrophages are associated with fatty liver disease through their effects on chronic inflammation, including cytokine and adipokine secretion (9). Previous studies reported that the status of inflammation associated with MAFLD might contribute to the cytokine ‘storm’, which further exacerbates the infection in patients with COVID-19 (10–12). The angiotensin-converting enzyme (ACE) 2 is the receptor for severe acute respiratory syndrome coronavirus 2 (SARS-CoV-2). An exploratory analysis found that ACE 2 mRNA expression in visceral fat (VF) positively correlated with BMI, and the engagement of SARS-CoV-2 on ACE 2 in the VF would impair the enzymatic activity of ACE 2 and enhance the production of inflammatory cytokines and their release into the systemic circulation (13). Therefore, we postulated the presence of MAFLD might exacerbate the virus-induced cytokine ‘storm’ associated with COVID-19, possibly through the hepatic release of multiple pro-inflammatory cytokines.

In this study, we investigated the association between peripheral blood IL-6 levels and MAFLD at admission and risk of having more severe illness in hospitalized patients with COVID-19.

## Materials and Methods

### Patients and Study Design

**Figure 1:**
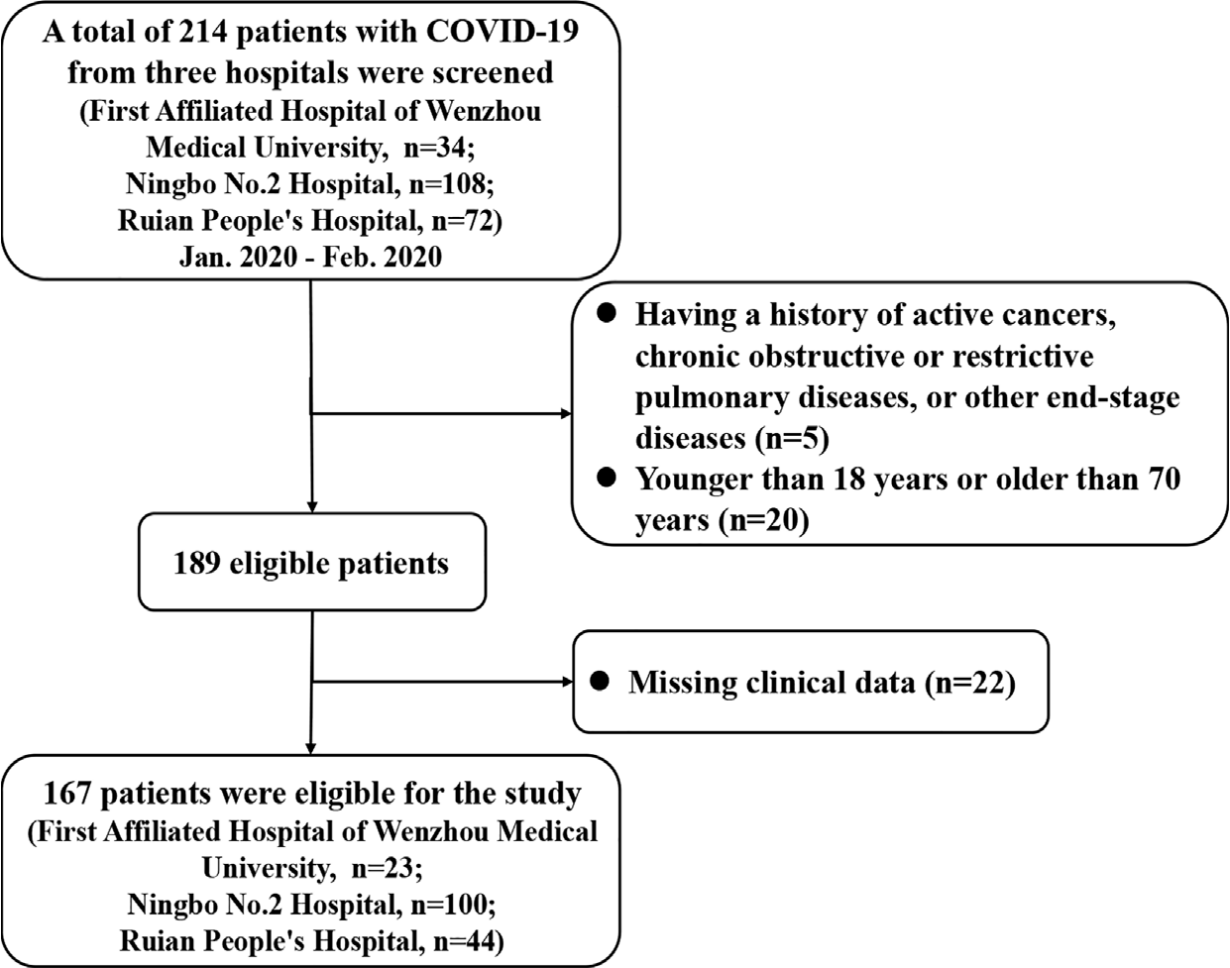
The flow chart for the study.

We enrolled 214 patients with laboratory-confirmed COVID-19 from three Chinese hospitals (the First Affiliated Hospital of Wenzhou Medical University, the Ningbo No.2 Hospital, and the Ruian People’s Hospital) between 17^th^ January and 11^th^ February 2020. As detailed in **Figure 1**, 47 subjects were excluded for the following reasons (1): younger than 18 years or older than 70 years; (2) having a history of active cancers, chronic obstructive or restrictive pulmonary diseases, or other end-stage diseases; (3) incomplete clinical/biochemical data. As a result of these exclusions, a sample of 167 patients was included in the final analysis. Some of these patients have been reported in a previous study (14). All patients had received standard treatments according to the Chinese COVID-19 management guidance.

The local ethics committees of the hospitals approved the study protocol (2020-002, 5 February 2020). All procedures performed involving the participants were in accordance with the ethical standards of the institutional research committee and with the 1964 Helsinki declaration. The requirement for written informed consent was waived due to the retrospective and anonymous nature of the study design.

### Laboratory and Clinical Data

Demographic and laboratory data, including age, sex, serum levels of alanine aminotransferase (ALT), aspartate aminotransferase (AST), gamma glutamyl-transpeptidase (GGT) and various cytokines were collected on the first day of hospital admission. In particular, circulating levels of IL-2, IL-4, IL-6, IL-10, tumor necrosis factor (TNF)-α, and interferon (IFN)-γ were measured using a flow cytometer (FACSCanto™ plus, America) and cytometric bead array techniques. The neutrophil-to-lymphocyte ratio (NLR), i.e., an established marker of systemic inflammation, was also calculated by dividing the absolute number of neutrophils by the absolute number of lymphocytes. FIB-4 score was calculated from the published formula (15).

Body mass index (BMI) was calculated using the formula weight (kilograms) divided by height (meters) squared. Overweight and obesity were defined, respectively, as BMI between 23 and 24.9 kg/m^2^ and BMI ≥ 25 kg/m^2^ in this Asian population. Type 2 diabetes mellitus (T2DM) was diagnosed as either self-reported history of disease, fasting glucose levels ≥7.0 mmol/L, hemoglobin A1c ≥6.5% (≥48 mmol/mol) or use of any anti-hyperglycemic drugs. Hypertension and dyslipidemia were diagnosed according to the consensus criteria (16).

In the whole cohort of patients, COVID-19 was diagnosed as a positive result by high-throughput sequencing or real-time reverse transcriptase-polymerase chain reaction assay of oropharyngeal swab specimens. Severity of COVID-19 was assessed during hospitalization and classified as two subtypes (severe and non-severe) based on the Chinese guidelines for management of COVID-19 (see **Supplementary Table 1**) (17).

### Liver Imaging

All patients were screened for fatty liver by computed tomography (CT). Liver and spleen measurements were performed using a CT post-processing workstation, and only one image (level of portal vein into the liver) of each patient was selected to complete attenuation measurements of liver and spleen. Three regions of interest in the liver were selected to avoid blood vessels, bile ducts and calcification. Two regions of interest in the spleen were selected at the same level. The respective means of the three attenuation values of the liver and two attenuation values of the spleen were calculated. Fatty liver was diagnosed by widely accepted CT characteristics, namely attenuation value of liver parenchyma (CTLP) <48 HU, or attenuation ratio of liver and spleen (LS ratio) <1.0, or attenuation difference of liver and spleen (LS diff.) <5 HU, respectively.

### MAFLD Definition

MAFLD was diagnosed as the presence of hepatic steatosis on CT scan and one of the following criteria: 1) overweight or obesity as defined by a BMI ≥23.0 kg/m^2^; 2) established T2DM; or 3) presence of at least two metabolic risk abnormalities (18). These metabolic risk abnormalities were defined as follows: 1) waist circumference ≥90/80 cm in men and women; 2) blood pressure ≥130/85 mmHg or drug treatment; 3) plasma triglycerides ≥1.70 mmol/L or drug treatment; 4) plasma HDL-cholesterol <1.03 mmol/L for men and <1.29 mmol/L for women or drug treatment; and 5) prediabetes (i.e., fasting glucose levels 5.6 to 6.9 mmol/L, or HbA1c 5.7% to 6.4%) (18).

### Statistical Analysis

Continuous variables are expressed as means ± SD or medians (inter-quartile ranges [IQR]) and compared using either the unpaired Student’s *t*-test or the Mann-Whitney test as appropriate. Categorical variables are expressed as numbers (percentages) and compared using the chi-squared test or the Fisher’s exact test as appropriate. The odds ratios (OR) and 95% confidence intervals (CIs) for the risk of having severe COVID-19 (as the outcome) associated with serum IL-6 levels and MAFLD at hospital admission were estimated using binary logistic regression analyses with adjustment for age, sex and metabolic co-morbidities (overweight/obesity, T2DM and hypertension). Interaction tests were used to evaluate associations between exposure variables and the outcome. Statistical analyses were two-sided and significance was set at *p* < 0.05. All statistical tests were performed using SPSS version 23.0 (SPSS Inc., Chicago, USA).

## Results

A cohort of 167 (42.5% men; mean age 49 years) hospitalized patients with laboratory-confirmed COVID-19 was included in the study. The median time from symptom onset to hospital admission was 9.0 (IQR 4.0–13.0) days. Forty-six (27.5%) of these patients had imaging-defined MAFLD and 32 (19.2%) patients were classified as having severe COVID-19. Among MAFLD patients, patients with severe COVID-19 had significantly higher FIB-4 score than those with non-severe COVID-19 (1.64 IQR [1.23–2.57] vs. 1.03 IQR [0.71–1.61], *p* = 0.047). The data about the cytokine levels is presented in the **Supplementary Table 2**.

**Figure 2:**
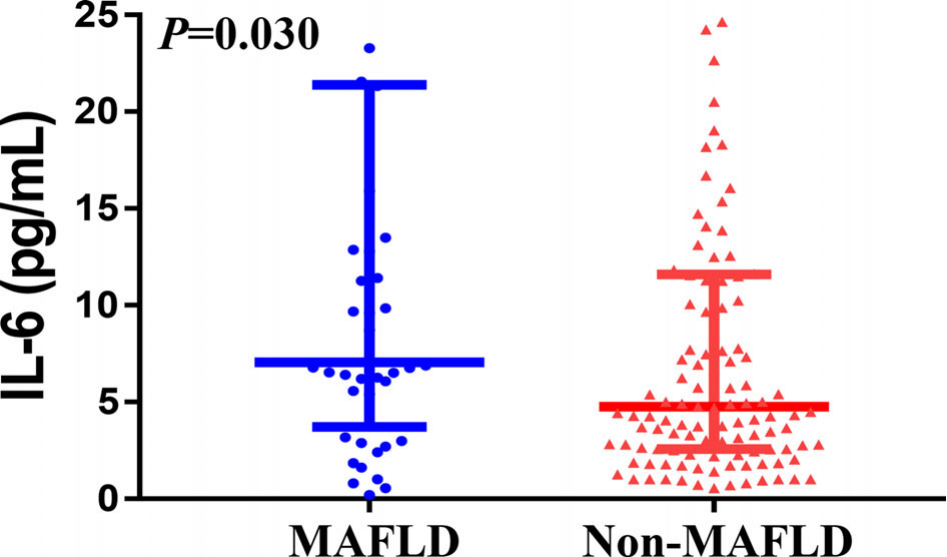
The association between IL-6 levels and MAFLD status among COVID-19 patients.

As shown in **Table 1**, patients with imaging-defined MAFLD had significantly higher levels at admission of serum IL-6 (median 7.1 [IQR, 4.3–20.0] vs. 4.8 [2.6–11.6] pg/mL, *p* = 0.030; **Figure 2**), liver enzymes and a higher proportion of overweight/obesity and dyslipidemia compared to those without MAFLD. After adjustment for age and sex, patients with MAFLD had an approximate 2.6-fold higher risk of severe COVID-19 than those without MAFLD (odds ratio 2.61, 95% CI 1.10–6.23, *p* = 0.030)).

**Table 1:** Baseline characteristics of COVID-19 patients, stratified by MAFLD status at hospital admission.

	Without MAFLD (n = 121)	With MAFLD (n = 46)	*P* value
**Demographics**			
Age, years	49.9 ± 13.2	47.7 ± 13.9	0.343
Male sex, n (%)	52 (43.0%)	19 (41.3%)	0.845
**Coexisting disorders**			
Type 2 diabetes, n (%)	15 (12.4%)	10 (21.7%)	0.131
Hypertension, n (%)	22 (18.2%)	13 (28.3%)	0.153
BMI ≥23 kg/m^2^, n (%)	75 (62.5%)	40 (87.0%)	**0.002**
Dyslipidemia, n (%)	85 (70.2%)	43 (93.5%)	**0.002**
**Laboratory parameters**			
WBC, x10^9^/L	4.8 (3.9–6.5)	5.0 (4.3–6.7)	0.458
Lymphocyte count, x10^9^/L	1.1 (0.8–1.4)	1.2 (0.9–1.6)	0.208
NLR	2.8 (1.8–4.9)	2.5 (1.7–3.6)	0.282
C-reactive protein, mg/L	11.5 (2.3–29.8)	17.2 (4.2–41.0)	0.122
Procalcitonin, ng/mL	0.03 (0.01–0.05)	0.03 (0.01–0.06)	0.474
D-dimer, mg/L	0.28 (0.14–0.66)	0.36 (0.20–0.59)	0.702
ALT, U/L	21.0 (15.0–31.0)	26.0 (20.0–39.8)	**0.024**
AST, U/L	22.0 (17.0–29.0)	27.0 (20.2–34.8)	**0.028**
GGT, U/L	24.0 (16.0–39.0)	31.0 (21.2–50.0)	**0.037**
TBIL, μmol/L	10.0 (6.6–14.0)	10.2 (7.5–14.0)	0.395
**Cytokines**			
IL-2, pg/mL	0.9 (0.6–1.4)	0.8 (0.5–1.4)	0.670
IL-4, pg/mL	1.3 (1.0–2.1)	1.0 (1.0–2.0)	0.518
IL-6, pg/mL	4.8 (2.6–11.6)	7.1 (4.3–20.0)	**0.030**
IL-10, pg/mL	2.6 (1.0–4.5)	3.6 (1.0–5.5)	0.251
TNF-α, pg/mL	1.1 (0.9–1.5)	1.0 (0.9–1.5)	0.306
IFN-γ, pg/mL	1.0 (1.0–1.6)	1.0 (0.9–1.9)	0.790
**Time from symptom onset to hospital admission, days**	9.0 (4.5–13.0)	7.0 (4.0–11.3)	0.267
**Severe COVID-19, n (%)**	19 (15.7%)	13 (28.3%)	0.065

Data are expressed as means ± SD, medians (IQRs) and number (percentages).

ALT, alanine aminotransferase; AST, aspartate transaminase; BMI, body mass index; GGT, gamma glutamyl-transpeptidase; IFN-γ, interferon gamma; IL, interleukin; MAFLD, metabolic dysfunction-associated fatty liver disease; NLR, Neutrophil-to-lymphocyte ratio; TBIL, total bilirubin; TNF-α, tumor necrosis factor-α; WBC, white blood cell. Bold values represent statistical significant P values.

As shown in **Table 2**, compared to those with non-severe COVID-19, patients with severe COVID-19 had significantly higher circulating levels of neutrophil-to-lymphocyte ratio, C-reactive protein, procalcitonin, D-dimer, IL-6 and IL-10 both in MAFLD and in non-MAFLD patients. Among non-MAFLD patients, those with severe COVID-19 were older, had significantly higher levels of WBC, liver enzymes (ALT, AST, GGT, and TBIL), and also had a higher proportion of T2DM, hypertension, and overweight/obesity than those with non-severe COVID-19. However, the significant differences in the aforementioned parameters were not observed among MAFLD patients, after stratification by severe COVID-19.

**Table 2:** Baseline characteristics of COVID-19 patients, stratified by both COVID-19 severity and MAFLD status.

	With MAFLD (n = 46)			Without MAFLD (n = 121)		
	Non-severe COVID-19 (n = 33)	SevereCOVID-19 (n = 13)	*P* value	Non-severe COVID-19 (n = 102)	SevereCOVID-19 (n = 19)	*P* value
**Demographics**						
Age, years	46.8 ± 14.1	50.2 ± 13.8	0.453	48.3 ± 13.3	58.2 ± 8.6	**0.003**
Male sex, n (%)	12 (36.4%)	7 (53.8%)	0.278	39 (38.2%)	13 (68.4%)	**0.015**
**Coexisting disorders**						
Type 2 diabetes, n (%)	9 (27.3%)	4 (30.8%)	0.813	10 (9.8%)	10 (52.6%)	**<0.001**
Hypertension, n (%)	8 (24.2%)	5 (38.5%)	0.335	15 (14.7%)	7 (36.8%)	**0.022**
BMI ≥23 kg/m^2^, n (%)	29 (87.9%)	11 (84.6%)	0.767	60 (58.8%)	15 (83.3%)	**0.048**
Dyslipidemia, n (%)	31 (93.9%)	12 (92.3%)	0.840	74 (72.5%)	11 (57.9%)	0.200
**Laboratory parameters**						
WBC, x10^9^/L	5.0 (3.9–6.4)	5.4 (4.8–7.2)	0.183	4.7 (3.7–5.9)	7.4 (5.4–10.2)	**<0.001**
Lymphocyte count, x10^9^/L	1.3 (0.9–1.7)	1.1 (0.9–1.2)	0.094	1.2 (0.9–1.6)	0.7 (0.5–0.8)	**<0.001**
NLR	2.5 (1.6–3.7)	10.2 (7.1–16.8)	**<0.001**	2.1 (1.6–3.1)	3.7 (3.2–4.4)	**0.012**
C-reactive protein, mg/L	9.7 (3.1–24.5)	47.6 (18.9–74.0)	**0.001**	9.2 (2.0–24.9)	32.0 (14.8–67.8)	**<0.001**
Procalcitonin, ng/mL	0.01 (0.01–0.04)	0.06 (0.04–0.08)	**0.013**	0.01 (0.01–0.04)	0.05 (0.04–0.07)	**<0.001**
D-dimer, mg/L	0.23 (0.12–0.34)	0.59 (0.47–1.21)	**0.002**	0.22 (0.13–0.30)	0.68 (0.55–0.83)	**<0.001**
ALT, U/L	30.0 (20.0–49.0)	22.0 (20.0–24.0)	0.241	20.0 (15.0–27.8)	26.0 (21.5–41.5)	**0.009**
AST, U/L	27.0 (19.0–35.0)	28.0 (25.0–34.0)	0.329	22.0 (17.0–28.0)	32.0 (21.5–40.5)	**0.003**
GGT, U/L	30.0 (20.0–51.0)	31.0 (24.0–47.0)	0.413	22.0 (15.2–32.8)	43.0 (27.5–84.5)	**<0.001**
TBIL, μmol/L	10.0 (7.1–13.6)	13.2 (9.3–14.0)	0.121	9.4 (6.4–13.6)	12.0 (9.2–18.5)	**0.033**
**Cytokines**						
IL-2, pg/mL	0.9 (0.5–1.6)	0.6 (0.5–1.1)	0.508	0.9 (0.5–1.5)	0.9 (0.7–1.0)	0.568
IL-4, pg/mL	1.0 (1.0–2.0)	1.0 (0.7–1.7)	0.267	1.4 (1.0–2.1)	1.2 (0.6–1.7)	0.058
IL-6, pg/mL	6.4 (2.9–9.8)	26.3 (12.9–45.4)	**<0.001**	4.6 (2.3–10.2)	4.9 (3.3–31.9)	**0.043**
IL-10, pg/mL	2.2 (1.0–4.2)	6.5 (4.3–12.5)	**<0.001**	2.3 (1.0–4.0)	4.8 (3.2–7.0)	**<0.001**
TNF-α, pg/mL	1.0 (1.0–1.5)	0.7 (0.2–1.4)	0.120	1.2 (1.0–1.6)	0.6 (0.2–1.5)	**0.007**
IFN-γ, pg/mL	1.0 (1.0–1.6)	1.4 (0.6–2.3)	0.834	1.0 (1.0–1.5)	1.3 (0.8–1.8)	0.439
**Time from symptom onset to hospital admission, days**	8.0 (4.0–12.0)	7.0 (5.5–9.5)	0.922	9.0 (4.0–13.0)	8.0 (5.0–10.0)	0.778

Data are expressed as means ± SD, medians (IQR) and number (percentages).

ALT, alanine aminotransferase; AST, aspartate transaminase; BMI, body mass index; GGT, gamma glutamyl-transpeptidase; IFN-γ, interferon gamma; IL, interleukin; MAFLD, metabolic associated fatty liver disease; NLR, Neutrophil-to-lymphocyte ratio; TBIL, total bilirubin; TNF-α, tumor necrosis factor-α; WBC, white blood cell. Bold values represent statistical significant P values.

**Figure 3:**
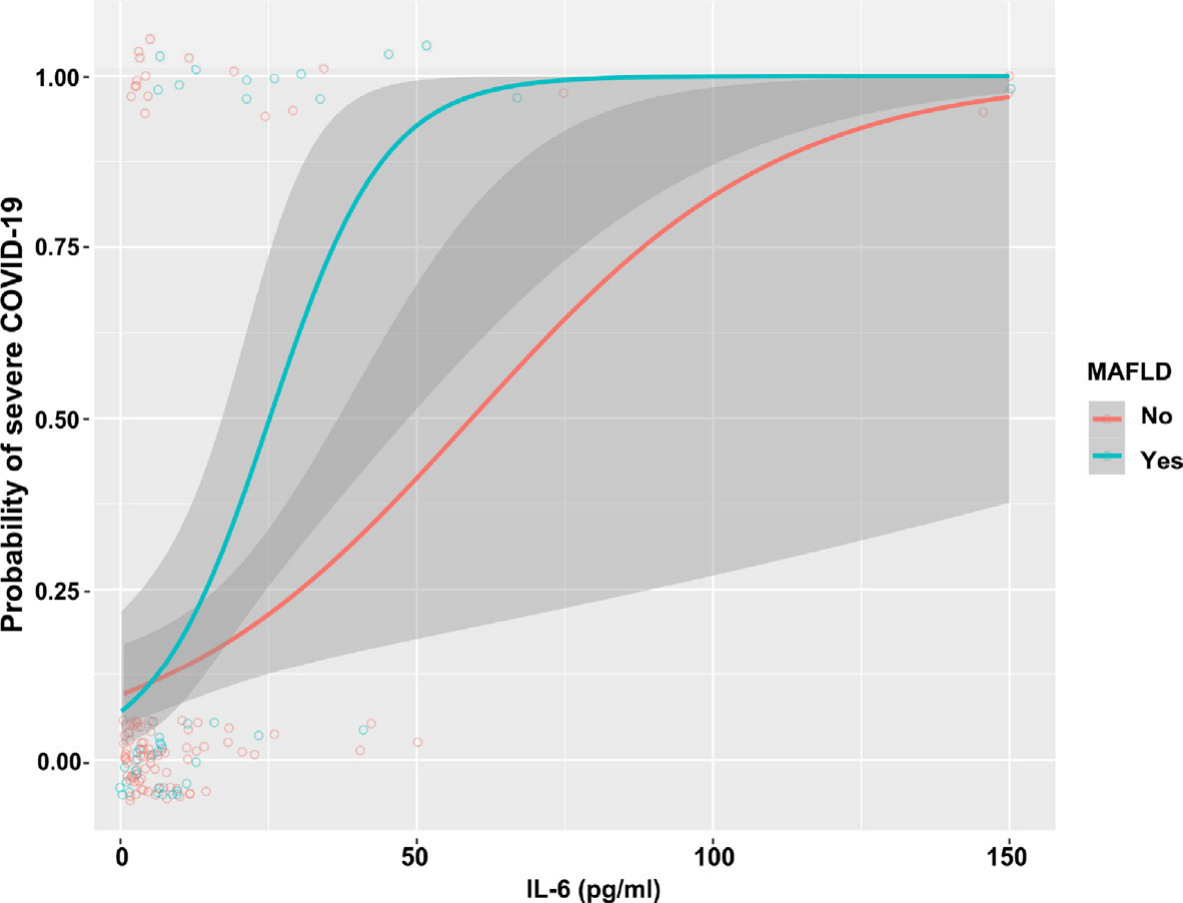
The association between increasing IL-6 levels and COVID-19 severity, stratified by MAFLD status.

As shown in **Table 3**, in the whole cohort of COVID-19 patients, there was a significant association between higher serum IL-6 levels and risk of having severe COVID-19 (unadjusted model: OR 1.06 [95% CI 1.03–1.09], *p*<0.001). Notably, this association remained significant even after adjustment for age, sex, overweight/obesity, T2DM, and hypertension (adjusted model 2: OR 1.04 [95% CI 1.02–1.09], *p* = 0.002). We also performed subgroup analyses and interaction tests to evaluate the existence of significant differences in serum IL-6 levels between patients with and without MAFLD at hospital admission, and to investigate interactions between exposure variables and the outcome. In the unadjusted model, elevated serum IL-6 levels were significantly associated with a higher risk of severe COVID-19 in both groups of patients. However, after adjustment for age and sex (adjusted model 1) and other metabolic co-morbidities (adjusted model 2), the significant association between elevated serum IL-6 levels and higher risk of severe COVID-19 persisted only in patients with MAFLD (adjusted model 2: OR 1.14 [95% CI 1.05–1.23], *p* = 0.002). The interaction test between IL-6 and MAFLD status on risk of severe COVID-19 was found to be statistically significant (*p-*value for interaction = 0.008). As shown in **Figure 3**, there was a clear dose-effect relationship between increasing values of IL-6 and the proportion of patients with severe COVID-19 in both MAFLD and non-MAFLD patients. Notably, elevated IL-6 levels had higher odds of severe COVID-19 among MAFLD patients than non-MAFLD patients.

**Table 3:** Association between serum IL-6 levels (as exposure) and severity of COVID-19 (as the outcome) in infected patients with and without MAFLD at hospital admission.

	Total (n = 167)		Without MAFLD (n = 121)		With MAFLD (n = 46)		*P* for interaction
	OR (95% CI)	*P value*	OR (95% CI)	*P value*	OR (95% CI)	*P value*	
Unadjusted Model	1.06 (1.03–1.09)	**<0.001**	1.04 (1.00–1.07)	**0.028**	1.11 (1.04–1.19)	**0.003**	0.055
Adjusted Model 1	1.05 (1.02–1.09)	**<0.001**	1.03 (0.99–1.06)	0.086	1.12 (1.04–1.21)	**0.004**	**0.022**
Adjusted Model 2	1.04 (1.02–1.09)	**0.002**	1.02 (0.99–1.05)	0.091	1.14 (1.05–1.23)	**0.002**	**0.008**

Data are expressed as odds ratio (OR) and 95% confidence intervals as tested by univariable (unadjusted) and multivariable (adjusted) logistic regression analysis.

Adjusted Model 1: adjusted for age and sex

Adjusted Model 2: adjusted for age, sex, overweight/obesity, type 2 diabetes, and hypertension. Bold values represent statistical significant P values.

**Figure 4:**
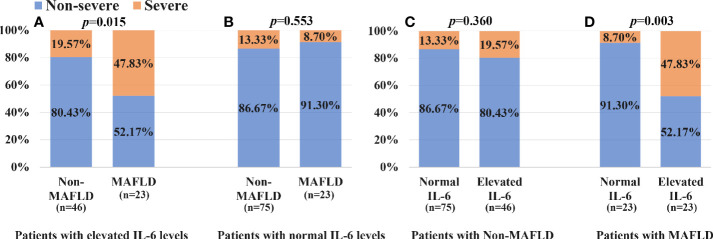
Association between IL-6 levels, MAFLD status and COVID-19 severity: **(A)** patients with elevated IL-6 levels; **(B)** patients with normal IL-6 levels; **(C)** patients with Non-MAFLD; **(D)** patients with MAFLD.

As shown in **Figure 4A**, patients with MAFLD and elevated IL-6 levels had a significantly higher proportion of severe COVID-19 than those with elevated IL-6 levels but without MAFLD (47.83% vs. 19.57%, *p* = 0.015). Among patients with normal IL-6 levels (**Figure 4B**), there was no significant difference in the percentage of severe COVID-19 between MAFLD and non-MAFLD patients (8.70% vs. 13.33%, *p* = 0.553). As shown in **Figure 4C**, among non-MAFLD patients, patients with elevated IL-6 levels appeared to have a higher proportion of severe COVID-19 than those with normal IL-6 levels but the effect was not statistically significant (19.57% vs. 13.33%, *p* = 0.360). There was a significantly lower proportion of patients with severe COVID-19 in MAFLD patients with normal IL-6 levels, than in those with elevated IL-6 levels (8.70% vs. 47.83%, *p* = 0.003; **Figure 4D**).

## Discussion

Our study shows for the first time that patients with laboratory-confirmed COVID-19 and imaging-defined MAFLD at hospital admission have significantly higher circulating IL-6 levels than their counterparts without MAFLD; increased IL-6 levels are also associated with higher odds of severe COVID-19; elevated IL-6 levels had higher odds of severe COVID-19 among MAFLD patients than non-MAFLD patients; and there is a significant interaction between elevated IL-6 levels and MAFLD with severe COVID-19. Notably, these significant associations persisted after adjusting for age, sex and coexisting metabolic co-morbidities. These results remind health care professionals caring for COVID-19 patients should be cognizant of the increased likelihood of severe COVID-19 in patients with elevated IL-6 levels, especially those with MAFLD.

It is known that serum IL-6 levels are elevated in metabolic syndrome, cardiovascular diseases and chronic inflammatory airways diseases (19). Wang et al. reported that fatty liver was independently associated with elevated IL-6 levels (20). However, the association between MAFLD and elevated IL-6 levels is currently uncertain. Our study showed that patients with MAFLD had higher levels of IL-6 than those without MAFLD.

Previous studies have shown that the inflammatory response plays an important role in COVID-19 severity (21, 22). The clinical deterioration observed in some infected patients has been related to the occurrence of a virus-induced cytokine ‘storm’ (23). Liu et al. have reported that the serum levels of IL-6 and C-reactive protein may effectively assess disease severity and predict adverse in-hospital outcomes in patients with COVID-19 (24). Besides, tocilizumab, i.e., a recombinant humanized monoclonal antibody IL-6 receptor inhibitor, has recently emerged as an alternative treatment for COVID-19 patients at risk of cytokine ‘storm’ (25). Accordingly, the results of the present study demonstrated that patients with severe COVID-19 had higher levels of several inflammatory biomarkers (e.g., NLR, CRP, procalcitonin, PCT, IL-6, and IL-10 levels).

Among COVID-19 patients, risk of severe infection and mortality are increased by co-morbidities such as obesity, T2DM, hypertension, cardiovascular disease, and cancer (26). Our previous studies showed that the presence of MAFLD in COVID-19 patients is associated with increased odds of severe COVID-19 (27, 28). A recent study by Petersen et al. also found that greater visceral adipose tissue accumulation was associated with a higher risk of ICU admission in COVID-19 patients (29). Since the diagnosis of MAFLD is based on the evidence of hepatic steatosis in addition to one or more metabolic disorders (namely overweight/obesity, presence of T2DM, or evidence of metabolic dysregulation), it is reasonable to hypothesize that the aggregation of these metabolic risk abnormalities in MAFLD may further aggravate the severity of COVID-19 (30, 31).

We do not know the exact underlying mechanisms by which MAFLD influences the association between elevated IL-6 levels and greater COVID-19 severity, but it is possible that the presence of MAFLD exacerbates the virus-induced cytokine ‘storm’, possibly through the hepatic release of multiple pro-inflammatory cytokines (including IL-6). Moreover, it is also possible to hypothesize that COVID-19 increases the risk of gut-derived endotoxemia, which may lead to macrophage activation and increased secretion of IL-6, thus further contributing to the virus-induced cytokine ‘storm’, especially in infected patients with coexisting abnormal liver function (9, 32). Thus, our findings further highlight the need for clinicians to pay close attention to COVID-19 patients with elevated serum IL-6 levels at hospital admission, especially those with coexisting MAFLD. Moreover, similar to liver, it is commonly to see COVID-19 affects the skeletal muscle such as loss of muscle mass, strength, and physical function (33). Skeletal muscle is also an essential source of IL-6 (34). Therefore, the COVID-19-muscle-IL-6 triangle is also worth investigating.

Our study has some limitations, including the relatively small sample size, the Asian ancestry of the cohort, and the lack of serial monitoring of circulating IL-6 levels during the hospital stay. Therefore, the results of our study will require further validation in larger cohorts of Asian and non-Asian patients with COVID-19. We excluded patients under the age of 18 years and over the age of 70 years, whether our research conclusions are applicable to these patients still needs to be verified. Moreover, sex hormone might affect the severity of MAFLD through the MyD88-dependent IL-6 signaling pathway (35), and recent studies highlighted the role of bio-immunological sex disparities underlying differences in the susceptibility to develop SARS-CoV-2 infection and its sequelae between females and males (36–38). Considering the MAFLD and COVID-19 are both sexually dimorphic and, therefore, additional and more extensive studies are needed to process data separately by sex (39). Finally, relying on CT imaging to diagnose fatty liver might misclassify mild MAFLD cases, and patients were recruited from three different Chinese hospitals, which might have introduced inter-individual variability in the assessment of MAFLD.

In conclusion, the results of this multicenter study show for the first time that there is an independent association and an interaction between serum IL-6 levels and MAFLD in hospitalized patients with severe COVID-19.

## Data Availability Statement

The raw data supporting the conclusions of this article will be made available by the authors, without undue reservation.

## Ethics Statement

The studies involving human participants were reviewed and approved by the ethics committees of the First Affiliated Hospital of Wenzhou Medical University, Ningbo No.2 Hospital, and Ruian People's Hospital. The ethics committees of the First Affiliated Hospital of Wenzhou Medical University, Ningbo No.2 Hospital, and Ruian People's Hospital waived the requirement of written informed consent for participation.

## Author Contributions

Study concept and design: FG, KZ, and M-HZ. Acquisition of data: H-DY, Q-FS, K-HP, T-YW, and Y-PC. Analysis and interpretation of data: FG and KZ. Drafting of the manuscript: FG and KZ. Critical revision of the manuscript for important intellectual content: GT, CB, and JG. Study supervision: M-HZ. All authors contributed to the article and approved the submitted version.

## Funding

This work was supported by grants from the National Natural Science Foundation of China (82070588), Medical Health Science and Technology Project of Zhejiang Provincial Health Commission (2018ZD039), Ruian Science and Technology Bureau (2020023), High Level Creative Talents from Department of Public Health in Zhejiang Province and Project of New Century 551 Talent Nurturing in Wenzhou. GT is supported in part by grants from the University School of Medicine of Verona, Verona, Italy. CB is supported in part by the Southampton NIHR Biomedical Research Centre (IS-BRC-20004), UK.

## Conflict of Interest

The authors declare that the research was conducted in the absence of any commercial or financial relationships that could be construed as a potential conflict of interest.

## Abbreviations

CI, confidence intervals; COVID-19, coronavirus disease-2019; IL, interleukin; OR, odds ratio; MAFLD, metabolic dysfunction-associated fatty liver disease; TBIL, total bilirubin.
