# Supplementary material for: Association and Interaction Between Serum Interleukin-6 Levels and Metabolic Dysfunction-Associated Fatty Liver Disease in Patients With Severe Coronavirus Disease 2019
Source: Front Endocrinol (Lausanne). 2021 Mar 8;12:604100. doi: 10.3389/fendo.2021.604100 (PMC7982673; doi:10.3389/fendo.2021.604100)
Supplement: Supplementary file 1 [file Table_1.docx]

**Supplementary Table 1. Clinical subtypes (mild, moderate, severe and critical) of COVID-19 severity according to the management guidelines.^1^**

| ***COVID-19 Severity*** | | ***Definition*** |
| --- | --- | --- |
| **Non-severe** | **Mild** | All clinical symptoms are mild and there are no signs of pneumonia on radiological imaging. |
|  | **Moderate** | Showing fever and respiratory symptoms with radiological findings of pneumonia |
| **Severe** | **Severe** | **Cases meeting any of the following criteria:**  (1) Respiratory distress (≥30 breaths/min);  (2) Oxygen saturation ≤93% at rest;  (3) Arterial partial pressure of oxygen (PaO_2_)/fraction of inspired oxygen (FiO_2_) ≤300mmHg (l mmHg = 0.133 kPa).  In high-altitude areas (at an altitude of over 1,000 meters above the sea level), PaO_2_/FiO_2_ shall be corrected by the following formula:  PaO_2_/FiO_2_ × [Atmospheric pressure (mmHg)/760]  Cases with chest imaging showing obvious lesion progression within 24-48 hours >50% shall be managed as severe cases |
|  | **Critically ill** | **Cases meeting any of the following criteria:**  (1) Respiratory failure requiring mechanical ventilation  (2) Shock  (3) Presence of any organ failure requiring ICU care |

11. National Health Commission & State Administration of Traditional Chinese Medicine. Diagnosis and Treatment Protocol for Novel Coronavirus Pneumonia (Trial Version 7). 2020 [EB/OL]. 2020.03.03.
