# Supplementary material for: Association and Interaction Between Serum Interleukin-6 Levels and Metabolic Dysfunction-Associated Fatty Liver Disease in Patients With Severe Coronavirus Disease 2019
Source: Front Endocrinol (Lausanne). 2021 Mar 8;12:604100. doi: 10.3389/fendo.2021.604100 (PMC7982673; doi:10.3389/fendo.2021.604100)
Supplement: Supplementary file 2 [file Table_2.docx]

**Supplementary Table 2.** **Difference analysis of cytokines between the two groups.**

|  | **Total** | **Non-severe** | **Severe** | ***P*-value** |
| --- | --- | --- | --- | --- |
| **IL-2, pg/mL** |  |  |  |  |
| Median (IQR) | 0.9 (0.5-1.4) | 0.9 (0.5-1.5) | 0.8 (0.5-1.1) | 0.326 |
| Mean (SD) | 1.3 (3.1) | 1.5 (3.5) | 0.9 (0.5) |  |
| **IL-4, pg/mL** |  |  |  |  |
| Median (IQR) | 1.2 (1.0-2.1) | 1.2 (1.0-2.1) | 1.1 (0.6-1.7) | 0.117 |
| Mean (SD) | 1.6 (0.9) | 1.6 (0.9) | 1.3 (1.00) |  |
| **IL-6, pg/mL** |  |  |  |  |
| Median (IQR) | 5.7 (2.8-12.7) | 5.0 (2.51-10.1) | 20.2 (4.6-37.3) | **<0.001** |
| Mean (SD) | 15.8 (40.4) | 8.2 (9.6) | 47.8 (83.9) |  |
| **IL-10, pg/mL** |  |  |  |  |
| Median (IQR) | 2.8 (1.0-4.6) | 2.2 (1.0-4.0) | 5.7 (3.9-8.0) | **<0.001** |
| Mean (SD) | 10.5 (83.9) | 3.1 (4.2) | 41.6 (190.8) |  |
| **TNF-α, pg/mL** |  |  |  |  |
| Median (IQR) | 1.0 (0.9-1.5) | 1.1 (1.0-1.6) | 0.7 (0.2-1.5) | 0.052 |
| Mean (SD) | 1.3 (1.2) | 1.4 (0.8) | 1.2 (2.1) |  |
| **IFN-γ, pg/mL** |  |  |  |  |
| Median (IQR) | 1.0 (1.0-1.6) | 1.0 (1.0-1.5) | 1.4 (0.7-1.9) | 0.404 |
| Mean (SD) | 2.7 (16.4) | 2.9 (18.1) | 2.1 (3.0) |  |
